# Evaluating the impact of glucokinase activation on risk of cardiovascular disease: a Mendelian randomisation analysis

**DOI:** 10.1186/s12933-022-01613-6

**Published:** 2022-09-23

**Authors:** Ke Wang, Mai Shi, Chuiguo Huang, Baoqi Fan, Andrea O. Y. Luk, Alice P. S. Kong, Ronald C. W. Ma, Juliana C. N. Chan, Elaine Chow

**Affiliations:** 1 Department of Medicine and Therapeutics, Prince of Wales Hospital, The Chinese University of Hong Kong, Shatin, Hong Kong Special Administrative Region China; 2 Li Ka Shing Institute of Health Sciences, Prince of Wales Hospital, The Chinese University of Hong Kong, Shatin, Hong Kong Special Administrative Region China; 3 Hong Kong Institute of Diabetes and Obesity, Prince of Wales Hospital, The Chinese University of Hong Kong, Shatin, Hong Kong Special Administrative Region China; 4 Phase 1 Clinical Trial Centre, Prince of Wales Hospital, The Chinese University of Hong Kong, Shatin, Hong Kong Special Administrative Region China

**Keywords:** Glucokinase, Glucokinase activator, Cardiovascular disease, Mendelian randomisation, Type 2 diabetes

## Abstract

**Background:**

Glucokinase activators (GKAs) are an emerging class of glucose lowering drugs that activate the glucose-sensing enzyme glucokinase (GK). Pending formal cardiovascular outcome trials, we applied two-sample Mendelian randomisation (MR) to investigate the impact of GK activation on risk of cardiovascular diseases.

**Methods:**

We used independent genetic variants in or around the *glucokinase* gene meanwhile associated with HbA_1c_ at genome-wide significance (*P* < 5 × 10^−8^) in the Meta-Analyses of Glucose and Insulin-related traits Consortium study (*N* = 146,806; European ancestry) as instrumental variables (IVs) to mimic the effects of GK activation. We assessed the association between genetically proxied GK activation and the risk of coronary artery disease (CAD; 122,733 cases and 424,528 controls), peripheral arterial disease (PAD; 7098 cases and 206,541 controls), stroke (40,585 cases and 406,111 controls) and heart failure (HF; 47,309 cases and 930,014 controls), using genome-wide association study summary statistics of these outcomes in Europeans. We compared the effect estimates of genetically proxied GK activation with estimates of genetically proxied lower HbA_1c_ on the same outcomes. We repeated our MR analyses in East Asians as validation.

**Results:**

Genetically proxied GK activation was associated with reduced risk of CAD (OR 0.38 per 1% lower HbA_1c_, 95% CI 0.29–0.51, *P* = 8.77 × 10^−11^) and HF (OR 0.54 per 1% lower HbA_1c_, 95% CI 0.41–0.73, *P* = 3.55 × 10^−5^). The genetically proxied protective effects of GKA on CAD and HF exceeded those due to non-targeted HbA_1c_ lowering. There was no causal relationship between genetically proxied GK activation and risk of PAD or stroke. The estimates in sensitivity analyses and in East Asians were generally consistent.

**Conclusions:**

GKAs may protect against CAD and HF which needs confirmation by long-term clinical trials.

**Supplementary Information:**

The online version contains supplementary material available at 10.1186/s12933-022-01613-6.

## Background

The increasing burden of diabetes is a major healthcare concern globally. The large proportion of patients with suboptimal glycaemic control [1] who are at high risk for multiple complications calls for novel treatment strategies. Glucokinase (GK) serves as a glucose sensor which converts glucose to glucose-6-phosphate, the first step towards ATP production [2]. The latter is essential for insulin secretion which promotes peripheral glucose uptake for cellular metabolism or storage as glycogen. Alpha-cell GK also suppresses glucose-regulated glucagon secretion [3]. The regulatory importance of GK is evidenced by maturity onset diabetes of the young type 2 (MODY2) due to inactivating mutations in this enzyme [4].

Many glucokinase activators (GKAs) have been designed and tested as new target-specific glucose-lowering drugs since 2003 [5]. These small molecules can bind to an allosteric site in the enzyme and facilitate GK activation by stabilizing a high-affinity conformation of GK to glucose. This allosteric site harbours most of the activating mutations implicated in genetic conditions such as congenital hyperinsulinism [5]. The binding of GKA to GK can improve the enzymatic kinetics and alter glucose sensitivity. Depending on the site of action, GKAs are further divided into dual-acting pancreatic and hepatic GKA and liver-selective GKA [6]. Within pancreatic β-cells, GKA facilitates glucokinase activation and enhances pancreatic glucose-stimulated insulin secretion. In the liver, GKA could activate glucokinase both directly and by dissociating the GK-GKRP (GK regulatory protein) complex to promote hepatic glucose uptake and glycogen synthesis [7].

Several GKAs have shown promising glucose-lowering effect in patients with type 2 diabetes. For example, dorzagliatin, a dual pancreatic and hepatic allosteric GKA lowered HbA_1c_ by 1.07% (95% confidence interval [CI]  − 1.19 to − 0.95) at a dose of 75 mg twice daily versus placebo which reduced HbA_1c_ by –0.50% (95% CI –0.68 to –0.32) during a 24-week of double-blind treatment in 463 drug-naive patients with type 2 diabetes in a phase 3 study [8]. Meanwhile, a liver-selective GKA, TTP-339, produced a placebo-subtracted 0.9% (95% CI − 1.5 to − 0.3%) reduction in HbA_1c_ over 6 months in 42 patients with type 2 diabetes at a dose of 800 mg once daily [9].

Cardiovascular diseases (CVDs) are major complications amongst patients with type 2 diabetes [10]. Early intensive blood-glucose lowering can protect diabetic populations from cardiovascular complications [11]. Nevertheless, glucose-lowering drugs may also act via other pleiotropic pathways that affect the cardiovascular safety, including those that are associated with an increased (e.g., rosiglitazone) or reduced (e.g., glucagon-like peptide-1 receptor agonists and sodium-glucose co-transporter-2 inhibitors) risk of CVD, as well as those that have neutral effects (e.g., dipeptidyl peptidase-4 inhibitors) [12].

Given the colossal cost in drug development and clinical trials, Mendelian randomisation (MR) provides a tool, where researchers can leverage genetic variants that are randomly allocated at conception as proxies to investigate the causal effect of an exposure on an outcome [13]. Moreover, MR studies are now increasingly applied to infer health effects of medications by proxying drug effects using variants located in the target genes [14]. Some examples include the use of *3-hydroxy-3-methylglutaryl-CoA reductase (HMGCR)* variants to mimic long-term treatment effects of HMGCR inhibitors (statins) [15] and the use of *glucagon like peptide 1 receptor (GLP1R)* variants to mimic that of GLP1R agonists [16].

With several GKAs in late phase clinical trials, in this study we utilized the MR framework to investigate the potential effects of GK activation by GKA on CVDs.

## Methods

### Study design

**Fig. 1:**
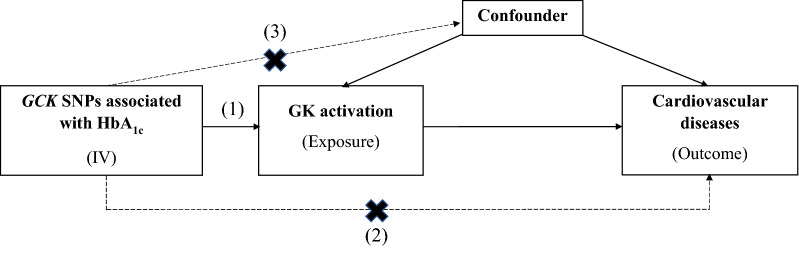
Directed acyclic graph of MR. MR is based on the three core assumptions: (1) IVs must be strongly associated with the exposure (typically *P* < 5 × 10^−8^); (2) IVs affect the outcome only through exposure and are not directly associated with the outcome; (3) IVs are not correlated with known exposure–outcome confounders. MR, Mendelian randomisation; IV, instrumental variable; SNP, single-nucleotide polymorphism; GK, glucokinase

Figure 1 and Additional file 1: Figure S1 illustrate the conceptual framework of our study. MR study is based on the three core assumptions: (1) instrumental variables (IVs) must be strongly associated with the exposure (typically *P* < 5 × 10^−8^); (2) IVs affect the outcome only through exposure and are not directly associated with the outcome; (3) IVs are not correlated with known exposure–outcome confounders. In the main analysis, we employed genetic variants located in or near the *GCK* gene as IVs of GK activation and evaluated the causal effects of genetically proxied GK activation on risk of CVDs using two-sample MR. We further performed MR analysis of genetically predicted lower HbA_1c_ as a proxy of non-targeted HbA_1c_ lowering, to assess whether the causal relevance of GKA and outcomes was contributed solely by its glucose-lowering effect. Throughout the study, we utilized the summary-level genetic data from published genome-wide association studies (GWAS) in MR analyses (Additional file 1: Table S1). All data used in the present work are publicly available and anonymized. All contributing studies had received appropriate ethical approval and patient consent.

### Identifying IVs for GK activation

The *GCK* gene encodes GK which is the protein target of GKA. For selection of IVs, we used single-nucleotide polymorphisms (SNPs) in or around *GCK* that were significantly associated with HbA_1c_ in the Meta-Analyses of Glucose and Insulin-related traits Consortium (MAGIC) study [17]. In the main analysis, we selected genetic variants within 100 kb downstream and upstream of *GCK* (genomic position on build GRCh37.p13: chromosome 7: 44182812–44229038) gene associated with HbA_1c_ (%, per 1% equals to per 11 mmol/mol) at a genome-wide level of statistical significance (*P* ≤ 5 × 10^−8^) and in low linkage disequilibrium (*r*^2^ < 0.3) in MAGIC. SNPs were selected by linkage disequilibrium clumping algorithm in PLINK (*r*^2^ threshold = 0.3, window size = 1000 kb) retaining the one with the smallest *P*-value. The 1000 Genomes European data were used as the reference for linkage disequilibrium estimation. The GWAS data used in the main analysis were restricted to those derived from subjects of European ancestry (*N* = 146,806) to avoid biases from population stratification.

### Outcome data

In this study, we focused on CVDs including coronary artery disease (CAD), peripheral arterial disease (PAD), stroke and heart failure (HF) as defined by the respective Consortiums in the original GWAS studies. We used the plasma insulin level and incident type 2 diabetes as positive controls given their expected associations with GKA use.

For CAD, we used summary statistics including 122,733 cases and 424,528 controls from a meta-analysis of CAD GWAS based on the CARDIOGRAMplusC4D Consortium and the UK BioBank [18]. Summary statistics for PAD (7098 cases and 206,541 controls) were obtained from the FinnGen Consortium. Summary statistics for stroke (40,585 cases and 406,111 controls) were obtained from the MEGASTROKE Consortium [19]. Summary statistics for HF (47,309 cases and 930,014 controls) were obtained from the Heart Failure Molecular Epidemiology for Therapeutic Targets (HERMES) Consortium [20]. Summary statistics for type 2 diabetes (12,931 cases and 57,196 controls) were obtained from the 70KforT2D study [21]. Summary statistics for plasma insulin level (3301 subjects) were obtained from a GWAS published by Sun et al. [22]. All participants were European ancestry and non-overlapping with those in the MAGIC. The detailed information of disease diagnoses and definitions can be found in the original publications or Consortium websites and were also briefly summarized in Additional file 1: Supplementary method.

### Sensitivity analysis

To assess the robustness of associations detected in the main analysis, we applied more stringent genetic variant selection criteria to exclude potential invalid instruments as sensitivity analyses. Specifically, we selected uncorrelated genetic variants (*r*^2^ threshold = 0.01, window size = 1000 kb, European 1000 Genome Project as reference panel) located completely in the gene body of *GCK* (genomic position on build GRCh37.p13: chromosome 7: 44182812–44229038) and were significantly associated with HbA_1c_ (*P* ≤ 5 × 10^−8^) in MAGIC (restricted to European ancestry). This was followed by two-sample MR analyses on the CVDs as previously defined.

### Replication in Asian population

We assessed the robustness of effect direction and statistical significance of the causal relationship between genetically proxied GK activation and CVD by repeating the same MR analyses in East Asians. We selected the IVs based on the associations derived from East Asian population (*N* = 33,307) in the MAGIC. For replication, we extracted SNPs within 100 kb downstream and upstream of *GCK* gene significantly associated (*P* ≤ 5 × 10^−8^) with HbA_1c_ (%) and in low linkage disequilibrium (*r*^2^ threshold = 0.3, window size = 1000 kb, East Asian 1000 Genome Project as reference panel). We collected the East-Asian-specific summary statistics of CVDs from Biobank Japan [23, 24].

### Comparing effects of genetically proxied GK activation with genetically predicted lower HbA_1c_ (non-targeted HbA_1c_ lowering)

To assess whether the cardiovascular effects of GK activation were different from those of non-targeted HbA_1c_ lowering, we also estimated the causal associations of non-targeted HbA_1c_ lowering with the outcomes. We used genetic variants associated with HbA_1c_ throughout the genome in addition to the *GCK* gene region in MAGIC (European ancestry, *P* ≤ 5 × 10^−8^, *r*^2^ threshold = 0.001, window size = 10000 kb, European 1000 Genome Project as reference panel) to mimic non-targeted HbA_1c_ lowering. The two-sample MR analyses were implemented on the outcomes with significant causal associations in the main analyses. We then estimated the magnitude of difference (β_diff_) by taking the difference between the MR beta coefficients for genetically proxied GK activation and genetically predicted lower HbA_1c_ (β_GK activation_ − β_Lower HbA1c_) [16]. The standard error for β_diff_ (SE_diff_) was derived using the propagation of error method as follows:

$${\text{ SE}}_{{\text{diff}}} = \sqrt {({\text{SE}}_{\text{GK activation}} )^2 + ({\text{SE}}_{{\text{Lower HbA}}1{\text{c }}} )^2 } ,$$

where SE_GK activation_ and SE_Lower HbA1c_ are the standard errors of the MR estimates for the associations of genetically proxied GK activation and genetically predicted lower HbA_1c_ with the respective outcomes.

### Statistical analysis

To validate the instrumental strength for MR analyses, we calculated the *F*-statistic to check if it exceeded the empirical threshold 10 [25]. We switched the effect allele of each variant to the HbA_1c_-decreasing allele to align with the expected effect of GKA. We harmonized the genetic associations of IVs with the exposures and outcomes by aligning effect alleles. We excluded palindromic variants with intermediate frequencies due to uncertainty in identifying the effect allele on the same strand in the two datasets [26]. The Wald ratio (the ratio of the genetic association with outcome to the genetic association of exposure) for each SNP was calculated.

We assessed the causal relationships by combining the Wald ratio using the random-effects inverse variance-weighted (IVW) method [27]. For the results indicating possible causal relationships in the IVW model, we performed additional analyses using sensitivity MR methods. These included Cochran’s *Q* statistic to measure heterogeneity among IVs [28], the weighted median method to provide consistent estimates if more than half of the genetic variants were valid IVs [29], the MR-Egger regression to detect horizontal pleiotropy [30], the MR Pleiotropy RESidual Sum and Outlier (MR-PRESSO) method to remove horizontal pleiotropy by detecting and correcting for potential outliers [31].

We carried out colocalization analysis to assess the validity of the instrumental variable assumptions, in case that the exposure and outcome might be causally influenced by distinct variants that happen to be in linkage disequilibrium [32]. We used the “coloc” package to quantify the probability of shared causal variants across exposure and outcomes that showed significant causality. This package uses approximate Bayes factor (ABF) computation to generate posterior probabilities (PP) with 5 exclusive hypotheses: (i) neither trait has a genetic association (PPH0); (ii) only trait 1 has a genetic association (PPH1); (iii) only trait 2 has a genetic association (PPH2); (iv) both traits are associated but with different causal variants (PPH3); (v) both traits are associated and share a single causal variant (PPH4). Colocalization analysis was performed by generating ± 100 kb windows from the *GCK* gene region.

All analyses were performed using R 4.1.2 software with the R packages “TwoSampleMR”, “MRPRESSO” and “coloc”. A *P*-value of < 0.05 was deemed statistically significant.

## Results

### Main analyses

We identified seventeen SNPs (*F-*statistic = 94) in or around *GCK* gene associated with HbA_1c_ (%) from MAGIC (European ancestry) as IVs for GK activation (Additional file 1: Table S2). As positive controls, we confirmed the genetically proxied GK activation were associated with higher plasma insulin level (beta 1.52 per 1% lower HbA_1c_, 95% CI 0.65–2.38, *P* = 0.0006) and lower risk of type 2 diabetes (odds ratio [OR] 0.09 per 1% lower HbA_1c_, 95% CI 0.05–0.17, *P* = 2.76 × 10^−15^; Table 1).

**Table 1:** Associations of genetically proxied GK activation with risks of T2D and insulin level

Exposure	Outcome	OR/Beta (95% CI)	*P*
*Main analysis*: Genetically proxied GK activation (per 1% lower HbA_1c_) instrumented by 17 SNPs	T2D	0.09 (0.05, 0.17)	2.76 × 10^−15^
	Insulin level	1.52 (0.65, 2.38)	0.0006
*Sensitivity analysis*: Genetically proxied GK activation (per 1% lower HbA_1c_) instrumented by 2 SNPs	T2D	0.11 (0.03, 0.35)	0.0002
	Insulin level	1.81 (−0.01, 3.64)	0.051

All estimations were based on the inverse variance weighted method. The population was restricted to European ancestry. 1% lower HbA_1c_ equals to 11 mmol/mol lower

OR, odds ratio; CI, confidence interval; T2D, type 2 diabetes; GK, glucokinase; SNP, single nucleotide polymorphism

**Fig. 2:**
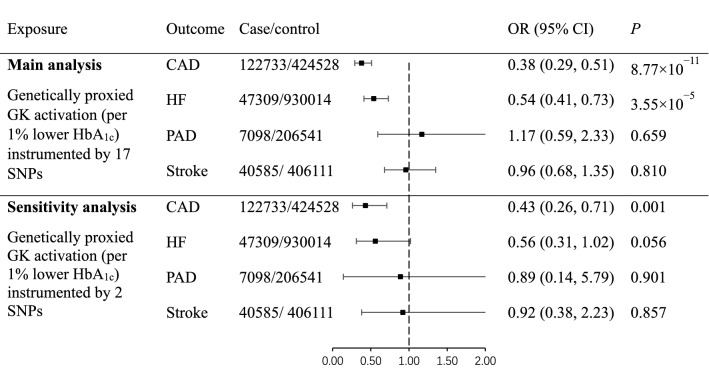
Associations of genetically proxied GK activation with risks of CAD, HF, PAD and stroke. The population was restricted to European ancestry. All estimations were based on the inverse variance weighted method. 1% lower HbA_1c_ equals to 11 mmol/mol lower. OR, odds ratio; CI, confidence interval; GK, glucokinase; SNP, single-nucleotide polymorphism; CAD, coronary artery disease; HF, heart failure; PAD, peripheral arterial disease

Genetically proxied GK activation were associated with decreased risk of CAD (OR 0.38 per 1% lower HbA_1c_, 95% CI 0.29–0.51, *P* = 8.77 × 10^−11^) and HF (OR 0.54 per 1% lower HbA_1c_, 95% CI 0.41–0.73, *P* = 3.55 × 10^−5^; Fig. 2). There was no significant heterogeneity in the IVW model (*P*_Heterogeneity_ = 0.292 for CAD, and 0.752 for HF) nor horizontal pleiotropy in MR-Egger regression (*P*_Egger-intercept_ = 0.067 for CAD, and 0.498 for HF; Table 2). No horizontal pleiotropy and outliers were detected in the MR-PRESSO model (*P*_Global-test_ = 0.266 for CAD, and 0.777 for HF). Results from weighted median model indicated that more than half of the genetic variants were valid IVs and the estimates were similar as those estimated by the IVW method (OR_weighted-median_ for CAD 0.39, 95% CI 0.27–0.57, *P*_weighted-median_ = 9.56 × 10^−7^; OR_weighted-median_ for HF 0.51, 95% CI 0.34–0.76, *P*_weighted-median_ = 8.62 × 10^−4^).

**Table 2:** Associations of genetically proxied GK activation with CAD and HF risks using different MR methods

Exposure	Method	OR (95% CI)	*P*
*Main analysis*: Genetically proxied GK activation (per 1% lower HbA_1c_) instrumented by 17 SNPs	CAD (122,733/424,528)		
	IVW (*P* for heterogeneity = 0.292)	0.38 (0.29, 0.51)	8.77 × 10^−11^
	Weighted median	0.39 (0.27, 0.57)	9.56 × 10^−7^
	MR-Egger regression	*P* for intercept = 0.067	
	MR-PRESSO (no outliers detected)	*P* for global test = 0.266	
	HF (47,309/930,014)		
	IVW (*P* for heterogeneity = 0.752)	0.54 (0.41, 0.73)	3.55 × 10^−5^
	Weighted median	0.51 (0.34, 0.76)	8.62 × 10^−4^
	MR-Egger regression	*P* for intercept = 0.498	
	MR-PRESSO (no outliers detected)	*P* for global test = 0.777	
*Sensitivity analysis*: Genetically proxied GK activation (per 1% lower HbA_1c_) instrumented by 2 SNPs	CAD (122,733/42,4528)		
	IVW (*P* for heterogeneity = 0.915)	0.43 (0.26, 0.71)	0.001
	Weighted median	NA	
	MR-Egger regression	NA	
	MR-PRESSO	NA	
	HF (47,309/930,014)		
	IVW (*P* for heterogeneity = 0.868)	0.56 (0.31, 1.02)	0.056
	Weighted median	NA	
	MR-Egger regression	NA	
	MR-PRESSO	NA	

The population was restricted to European ancestry. 1% lower HbA_1c_ equals to 11 mmol/mol lower

OR, odds ratio; CI, confidence interval; GK, glucokinase; SNP, single-nucleotide polymorphism; CAD, coronary artery disease; HF, heart failure; IVW, inverse variance weighted; MR-PRESSO, MR Pleiotropy RESidual Sum and Outlier; NA, not applicable

We performed colocalization analysis to assess potential confounding due to linkage disequilibrium (Additional file 1: Table S3). The posterior probability that genetically proxied GK activation and CAD or HF shared different causal variants was low (PPH3 = 0.052 for CAD; PPH3 = 0.017 for HF), though we also did not have enough evidence to support colocalization (PPH4 = 0.190 for CAD; PPH4 = 0.048 for HF). Of note, the probability of [PPH4/(PPH3 + PPH4)] which represents the probability of colocalization conditional on the presence of a causal variant for outcomes provided some evidence for colocalization (0.785 for CAD; 0.738 for HF).

We did not observe causal relationship between genetically proxied GK activation and PAD (OR 1.17, 95% CI 0.59–2.33, *P* = 0.659) or stroke (OR 0.96, 95% CI 0.68–1.35, *P* = 0.810; Fig. 2).

### Sensitivity analyses

As sensitivity analyses, we identified two uncorrelated *GCK* variants (*F*-statistic = 207) significantly associated with HbA_1c_ (%) in MAGIC (European ancestry) as IVs for GK activation (Additional file 1: Table S4). The results of sensitivity analyses were overall consistent with those of main analyses. GK activation proxied by the two variants remained significantly associated with reduced risk of CAD (OR 0.43, 95% CI 0.26–0.71, *P* = 0.001) and the association was slightly attenuated for HF (OR 0.56, 95% CI 0.31–1.02, *P* = 0.056; Fig. 2). There was no heterogeneity in the IVW model (*P*_Heterogeneity_ = 0.915 for CAD, and 0.466 for HF). We did not observe causal relationship in sensitivity analyses between genetically proxied GK activation and PAD (OR 0.89, 95% CI 0.14–5.79, *P* = 0.901) or stroke (OR 0.92, 95% CI 0.38–2.23, *P* = 0.857; Fig. 2).

### Replication analyses in Asian population

For replication in Asian population, we identified three SNPs (*F*-statistic = 56) associated with HbA_1c_ (%) as the IVs (Additional file 1: Table S5). GK activation proxied by the three SNPs were significantly associated with reduced risk of CAD (OR 0.47, 95% CI 0.28–0.80, *P* = 0.005). The estimates of HF risk (OR 0.67, 95% CI 0.22–2.08, *P* = 0.493) was directionally consistent with the results in European population, albeit not significant due to fewer cases in Biobank Japan (Additional file 1: Table S6). No causal relationship was observed between genetically proxied GK activation and PAD (OR 1.01, 95% CI 0.27–3.83, *P* = 0.987) or stroke (OR 0.84, 95% CI 0.44–1.59, *P* = 0.594; Additional file 1: Table S6).

### Comparisons of genetically proxied GK activation with genetically predicted lower HbA_1c_ (non-targeted HbA_1c_ lowering)

**Fig. 3:**
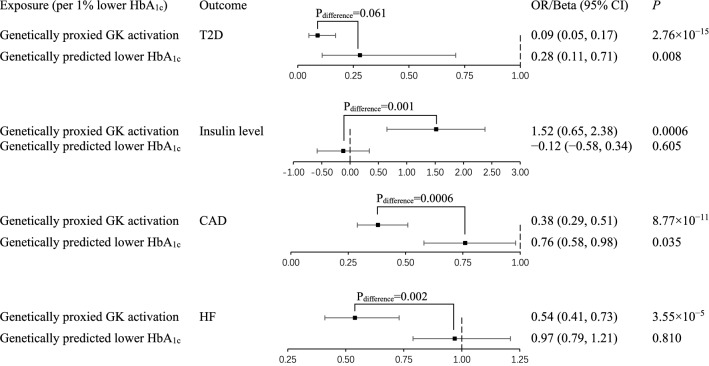
Comparisons of effects of genetically proxied GK activation and genetically predicted lower HbA_1c_. All estimations were based on the inverse variance weighted method. The population was restricted to European ancestry. 1% lower HbA_1c_ equals to 11 mmol/mol lower. OR, odds ratio; CI, confidence interval; GK, glucokinase; SNP, single-nucleotide polymorphism; T2D, type 2 diabetes; CAD, coronary artery disease; HF, heart failure

We identified 75 genome-wide SNPs (Additional file 1: Table S7) associated with HbA_1c_ in MAGIC (European ancestry, *P* ≤ 5 × 10^−8^, *r*^2^ < 0.001) as IVs for non-targeted HbA_1c_ lowering (Fig. 3). Genetically predicted lower HbA_1c_ was associated with a decreased risk of type 2 diabetes (OR 0.28 per 1% lower HbA_1c_, 95% CI 0.11–0.71, *P* = 0.008) and the effect size was smaller in magnitude than the estimate of genetically proxied GK activation, albeit not significant (*P*_difference_ = 0.061). There was no causal relationship between genetically predicted lower HbA_1c_ and insulin level (beta 1.52 [0.65, 2.38] versus − 0.12 [− 0.58, 0.34] per 1% lower HbA_1c_, *P*_difference_ = 0.001), confirming the validity of the IVs selected for GK activation. Though genetically predicted lower HbA_1c_ also reduced risk of CAD (OR 0.76 per 1% lower HbA_1c_, 95% CI 0.58–0.98, *P* = 0.035), the risk-reducing effect size for CAD by genetically proxied GK activation was two-fold larger than that of genetically predicted lower HbA_1c_ (*P*_difference_ = 0.0006). We did not observe causal relationship between genetically predicted lower HbA_1c_ and HF (OR 0.97 per 1% lower HbA_1c_, 95% CI 0.79–1.21, *P* = 0.810). We observed similar results after excluding the *GCK* variants (rs2908277 and rs2971670) from the 75 SNPs that mimicked non-targeted HbA_1c_ lowering (Additional file 1: Table S8).

## Discussion

In this study, we investigated the causal relationships between GK activation and CVDs using MR approach. We provided genetic evidence inferring that GK activation by GKA treatment may reduce risks of CAD and HF. Upon comparison, the effect of GK activation exceeded that proxied by non-targeted HbA_1c_ lowering. Taken together, our findings suggested that GK-targeted glucose-lowering medications may have stronger protective effects on CAD and HF than non-targeted glucose-lowering regimens. Our results provide the premise for designing RCTs of GKA in cardiovascular protection.

Since GKAs are not yet available in the market, there are no observational studies to support the potential long-term effects of GKA treatment. Compared with observational studies, MR design is less susceptible to confounding and time-related biases by leveraging the random allocation of genetic variants at conception by nature. Although we found genetically predicted lower HbA_1c_ was also associated with reduced risk of CAD which was in line with previous MR analysis [33], there is heterogeneity regarding the cardiovascular effects of glucose-lowering drugs in clinical trials. Not all glucose-lowering drugs prevented these complications and some thiazolidinediones were associated with increased cardiovascular risk [12].

Of note, some of GKAs had shown adverse effects in clinical trials. One is higher incidence of hypoglycaemia events due to overstimulation of pancreatic GK, which affect hepato-selective GKAs to a lesser extent. Hypoglycemia has been associated with an increased risk of cardiovascular events [34] via a number of potential mechanisms [35]. In 52-week trials of the dual-acting GKA dorzagliatin in drug-naïve and metformin treated Chinese patients with type 2 diabetes versus placebo, the reported incidence of clinically significant hypoglycaemia (< 3.0 mmol/L) was low (0.3% and 0.8% respectively) with no severe hypoglycaemic events [8, 36]. Overstimulation of hepatic GK may lead to hypertriglyceridemia [37, 38], which could increase the risk of CVDs. In phase 3 trials of dorzagliatin among metformin-treated T2D patients, hypertriglyceridemia related to the study drug was higher in the intervention group versus placebo (2 vs. 0.5%) [36]. Overall, the potential adverse effects of GKA on hypoglycemia and lipids may depend on the degree and balance of pancreatic and hepatic GK activation.

Our study had several strengths. We applied different MR sensitivity methods to test the robustness of our findings against different MR assumptions and replicated our findings in European and East Asian populations. However, we acknowledge certain limitations and recommend that our results should be interpreted with appropriate caution. First, although our work provided a robust indication of significance and effect direction, the effect estimates reported in this study do not directly reflect the clinical effect sizes or health impacts of GKA treatment on cardiovascular events. The magnitude by which the reported effect estimates approximated true effects would depend on the degree by which genetically-proxied GK activation mimicked the true GKA effect on HbA_1c_ reduction. Moreover, compared to RCTs that investigate short-term pharmacological treatment.

GKAs include dual-acting pancreatic and hepatic GKA and liver-selective GKA. In the current study, we were not able to select organ-specific *GCK* variants associated with HbA_1c_ to distinguish the effects of pancreatic and hepatic GK activation. Therefore, our results might better proxy dual-acting GKA than the liver-selective GKA. In response to rising blood glucose, hepatic GK can be released from GK-GKRP complex for glucose uptake and glycogen synthesis in the liver [39]. It has been reported that GKA can influence the interaction of GK and GKRP and enhance GK translocation [7]. To this end, analysis of genetically predicted HbA_1c_/glucose reduction instrumented by *GCKR* SNPs might provide better approximation to mimic liver-selective GKA. However, we were not able to identify any *GCKR* SNPs that were associated with HbA_1c_ at a genome-wide level of statistical significance in the MAGIC. Of note, studies have shown a common functional variant in *GCKR* (rs1260326) associated with increased translocation of GK from GK-GKRP complex, lower fasting plasma glucose, but with increased de novo lipogenesis, non-alcoholic fatty liver disease (NAFLD) and higher serum triglycerides [40], as well as an increased risk of CAD [41]. Therefore, *GCKR* SNPs should be also considered in some way in the future analyses.

We did not observe causal relationships between genetically proxied GK activation and PAD or stroke, which could be due to few number of events in the original GWAS, an absence of true causal associations or the bias in our IV selection. We only proxied the glycaemic effects of GKAs using the HbA_1c_-associated SNPs in *GCK*, but GKAs might exert effects on CVD through other mechanisms which were not fully captured by our selected IVs. In addition, MR studies of possible drug effects may be subject to selection bias due to inclusion of survivors with certain genetic make-up of the outcomes under investigation. MR studies usually utilise GWAS recruited in mid-life well after the random allocation of genetic variants at conception. Therefore, we were not able to exclude potential selection bias from competing risk before recruitment for diseases which share the same risk factors that typically occur at younger ages, which may attenuate the effect estimates in the analyses.

Finally, our study was different from a drug-target MR analysis, where the primary aim was to assess whether perturbation of the protein (or functional protein) level of certain drug target could affect the outcomes [42]. This is mainly because GKAs activate GK by changing the conformation of the enzyme rather than regulating its protein expression. Nevertheless, a drug-target MR on *GCK* or *GKRP* warrants future investigation to explore the alternative druggable potentials of the GK-related pathways for treating both diabetes and cardiovascular complications.

## Conclusions

In conclusion, we provided genetic evidence suggesting that GK activation by GKA treatment might reduce risks of CAD and HF. Patients with type 2 diabetes at high risk of cardiovascular complications may benefit from this novel glucose-lowering drug although RCTs are required to confirm these findings.

## Supplementary Information

**Additional file 1.** **Table S1.** Information of included summary-level statistics. **Table S2.** Associations of instrumental variables for GK activation in main analyses with exposure and outcomes. **Table S3.** Colocalization analysis of genetically proxied GK activation and outcomes. **Table S4.** Associations of instrumental variables for GK activation in sensitivity analyses with exposure and outcomes. **Table S5.** Instrumental variables for GK activation in East Asian population and their associations with HbA1c. **Table S6.** Associations of genetically proxied GK activation with risks of cardiovascular outcomes in East Asian population. ** Table S7.** Instrumental variables for non-targeted HbA1c lowering and their associations with HbA1c. **Table S8.** Associations of genetically predicted lower HbA1c with outcomes after removing GCK variants. **Figure S1.** Conceptual framework of study design. **Supplementary Method. **Brief summary of outcome definition.

## Data Availability

All summary statistics used in the present work are publicly available, and can be accessed and downloaded through websites listed in Table S1.

## Abbreviations

CAD: Coronary artery disease
CVD: Cardiovascular disease
GK: Glucokinase
GKA: Glucokinase activator
GKRP: Glucokinase regulatory protein
GLP1R: Glucagon like peptide 1 receptor
GWAS: Genome-wide association studies
HF: Heart failure
HMGCR: 3-Hydroxy-3-methylglutaryl-CoA reductase
IV: Instrumental variable
IVW: Inverse variance-weighted
MAGIC: Meta-Analyses of Glucose and Insulin-related traits Consortium
MR: Mendelian randomisation
MR-PRESSO: MR Pleiotropy RESidual Sum and Outlier
PAD: Peripheral arterial disease
