# Supplementary material for: Evaluating the impact of glucokinase activation on risk of cardiovascular disease: a Mendelian randomisation analysis
Source: Cardiovasc Diabetol. 2022 Sep 23;21:192. doi: 10.1186/s12933-022-01613-6 (PMC9503210; doi:10.1186/s12933-022-01613-6)
Supplement: Supplementary file 1 — Additional file 1. Table S1. Information of included summary-level statistics. Table S2. Associations of instrumental variables for GK activation in main analyses with exposure and outcomes. Table S3. Colocalization analysis of genetically proxied GK activation and outcomes. Table S4. Associations of instrumental variables for GK activation in sensitivity analyses with exposure and outcomes. Table S5. Instrumental variables for GK activation in East Asian population and their associations with HbA1c. Table S6. Associations of genetically proxied GK activation with risks of cardiovascular outcomes in East Asian population. Table S7. Instrumental variables for non-targeted HbA1c lowering and their associations with HbA1c. Table S8. Associations of genetically predicted lower HbA1c with outcomes after removing GCK variants. Figure S1. Conceptual framework of study design. Supplementary Method. Brief summary of outcome definition. [file 12933_2022_1613_MOESM1_ESM.docx]

**Supplementary tables**

Table S1. Information of included summary-level statistics.

| Trait or disease | Consortium or cohort study | Participants | Web source and publication |
| --- | --- | --- | --- |
| HbA_1c_ | Meta-Analyses of Glucose and Insulin-related traits Consortium (MAGIC) | 146806 European-descent individuals | <https://magicinvestigators.org/downloads/>  PMID: 34059833 |
| Coronary artery disease | Meta analysis of CARDIOGRAMplusC4D Consortium and UK BioBank | 122733 cases and 424528 controls of European ancestry | <https://www.ebi.ac.uk/gwas/studies/GCST005195>  PMID: 29212778 |
| Peripheral arterial disease | FinnGen Consortium | 7098 cases and 206541 controls of European ancestry (Data freeze 2) | <https://www.finngen.fi/en> |
| Stroke | MEGASTROKE consortium | 40585 cases and 406111 controls of European ancestry | <https://www.megastroke.org/>  PMID: 29531354 |
| Heart failure | Heart Failure Molecular Epidemiology for Therapeutic Targets (HERMES) Consortium | 47309 cases and 930014 controls of European ancestry | <https://www.hermesconsortium.org/>  PMID: 31919418 |
| Type 2 diabetes | 70KforT2D study | 12931cases and 57196 controls of European ancestry | <https://www.ebi.ac.uk/gwas/studies/GCST005413>  PMID: 29358691 |
| Plasma insulin | INTERVAL study | 3301 European-descent individuals | <https://www.ebi.ac.uk/gwas/studies/GCST005806>  PMID: 29875488 |
| HbA_1c_ | Meta-Analyses of Glucose and Insulin-related traits Consortium (MAGIC) | 33307 East Asian-descent individuals | <https://magicinvestigators.org/downloads/>  PMID: 34059833 |
| Coronary artery disease | BioBank Japan | 29319 cases and 183134 controls of East Asian ancestry | <http://jenger.riken.jp/en/>  PMID: 32514122 |
| Heart failure | BioBank Japan | 10540 cases and 168186 controls of East Asian ancestry | https://pheweb.jp/pheno/  PMID: 34594039 |
| Peripheral arterial disease | BioBank Japan | 3593 cases and 208860 controls of East Asian ancestry | <http://jenger.riken.jp/en/>  PMID: 32514122 |
| Ischemic stroke | BioBank Japan | 17671 cases and 192383 controls of East Asian ancestry | <http://jenger.riken.jp/en/>  PMID: 32514122 |

Table S2. Associations of instrumental variables for GK activation in main analyses with exposure and outcomes.

| SNP | Position | Effect allele | Other allele | EAF | Effect size for HbA_1c_ | *P* for HbA_1c_ | Effect size for CAD | *P* for CAD | Effect size for HF | *P* for HF | Effect size for PAD | *P* for PAD | Effect size for stroke | *P* for stroke |
| --- | --- | --- | --- | --- | --- | --- | --- | --- | --- | --- | --- | --- | --- | --- |
| rs2595701 | 7:44148553 | A | G | 0.317 | 0.010 | 4.8E-11 | -0.018 | 0.02 | -0.019 | 0.03 | 0.009 | 0.65 | 0.000 | 0.97 |
| rs61736256 | 7:44153614 | C | G | 0.964 | -0.023 | 3.0E-09 | -0.010 | 0.57 | -0.024 | 0.26 | NA | NA | 0.000 | 0.99 |
| rs2908277 | 7:44183433 | A | G | 0.117 | 0.017 | 1.3E-18 | -0.015 | 0.15 | -0.002 | 0.87 | 0.024 | 0.35 | 0.014 | 0.29 |
| rs28684786 | 7:44206824 | T | C | 0.901 | 0.020 | 8.1E-10 | -0.015 | 0.21 | -0.022 | 0.11 | -0.034 | 0.22 | 0.010 | 0.59 |
| rs1303722 | 7:44219074 | T | C | 0.463 | -0.012 | 1.9E-23 | 0.022 | 0.001 | 0.008 | 0.29 | 0.004 | 0.84 | 0.000 | 0.99 |
| rs2300584 | 7:44219338 | A | G | 0.751 | -0.023 | 1.2E-55 | 0.030 | 0.0003 | 0.014 | 0.15 | -0.019 | 0.37 | -0.002 | 0.83 |
| rs2284769 | 7:44222220 | C | G | 0.889 | 0.013 | 7.5E-09 | -0.019 | 0.09 | 0.009 | 0.50 | 0.024 | 0.35 | -0.002 | 0.89 |
| rs2971670 | 7:44226101 | T | C | 0.181 | 0.032 | 5.1E-88 | -0.026 | 0.004 | -0.022 | 0.04 | -0.023 | 0.45 | -0.009 | 0.44 |
| rs74897641 | 7:44230381 | A | G | 0.013 | 0.047 | 3.0E-11 | 0.018 | 0.52 | -0.014 | 0.68 | 0.114 | 0.25 | -0.015 | 0.76 |
| rs3757840 | 7:44231216 | T | G | 0.52 | 0.022 | 4.3E-71 | -0.026 | 0.0002 | -0.022 | 0.01 | -0.012 | 0.52 | 0.001 | 0.95 |
| rs138917529 | 7:44235694 | A | T | 0.983 | 0.038 | 9.5E-11 | -0.073 | 0.02 | -0.001 | 0.98 | -0.039 | 0.46 | 0.091 | 0.03 |
| rs79784692 | 7:44256526 | A | G | 0.933 | 0.025 | 8.2E-15 | -0.009 | 0.53 | 0.003 | 0.83 | 0.037 | 0.31 | -0.016 | 0.41 |
| rs2075067 | 7:44263028 | A | C | 0.15 | 0.011 | 1.5E-08 | -0.011 | 0.27 | -0.006 | 0.61 | 0.023 | 0.35 | 0.004 | 0.75 |
| rs10951758 | 7:44266077 | A | G | 0.526 | -0.013 | 6.5E-20 | 0.013 | 0.08 | 0.009 | 0.27 | 0.025 | 0.21 | 0.007 | 0.50 |
| rs59374739 | 7:44266570 | T | G | 0.727 | -0.018 | 6.8E-22 | NA | NA | 0.019 | 0.20 | NA | NA | 0.009 | 0.52 |
| rs4724295 | 7:44300964 | A | G | 0.627 | 0.009 | 3.1E-09 | -0.013 | 0.07 | -0.006 | 0.48 | 0.024 | 0.20 | -0.012 | 0.27 |
| rs66482342 | 7:44319048 | T | C | 0.086 | 0.018 | 1.4E-09 | 0.003 | 0.83 | -0.017 | 0.28 | 0.040 | 0.23 | 0.001 | 0.98 |

The population was restricted to European ancestry. EAF were from Meta-Analyses of Glucose and Insulin-related traits Consortium. GK, glucokinase; SNP, single nucleotide polymorphism; EAF, effect allele frequency. CAD, coronary artery disease; HF, heart failure; PAD, peripheral arterial disease.

Table S3. Colocalization analysis of genetically proxied GK activation and outcomes.

| Exposure | Outcome | PPH0 | PPH1 | PPH2 | PPH3 | PPH4 |
| --- | --- | --- | --- | --- | --- | --- |
| Genetically proxied GK activation | CAD | 0.000 | 0.758 | 0.000 | 0.052 | 0.190 |
|  | HF | 0.000 | 0.935 | 0.000 | 0.017 | 0.048 |

The population was restricted to European ancestry. GK, glucokinase; PP, posterior probability; CAD, coronary artery disease; HF, heart failure.

Table S4. Associations of instrumental variables for GK activation in sensitivity analyses with exposure and outcomes.

| SNP | Position | Effect allele | Other allele | EAF | Effect size for HbA_1c_ | *P* for HbA_1c_ | Effect size for CAD | *P* for CAD | Effect size for HF | *P* for HF | Effect size for PAD | *P* for PAD | Effect size for stroke | *P* for stroke |
| --- | --- | --- | --- | --- | --- | --- | --- | --- | --- | --- | --- | --- | --- | --- |
| rs2908277 | 7:44183433 | A | G | 0.117 | 0.017 | 1.3E-18 | -0.015 | 0.15 | -0.002 | 0.87 | 0.024 | 0.35 | 0.014 | 0.29 |
| rs2971670 | 7:44226101 | T | C | 0.181 | 0.032 | 5.1E-88 | -0.026 | 0.004 | -0.022 | 0.04 | -0.023 | 0.45 | -0.009 | 0.44 |

The population was restricted to European ancestry. EAF were from Meta-Analyses of Glucose and Insulin-related traits Consortium. GK, glucokinase; SNP, single nucleotide polymorphism; EAF, effect allele frequency. CAD, coronary artery disease; HF, heart failure; PAD, peripheral arterial disease.

Table S5. Instrumental variables for GK activation in East Asian population and their associations with HbA_1c_.

| SNP | Position | Effect allele | Other allele | EAF | Effect size | Standard error | *P* |
| --- | --- | --- | --- | --- | --- | --- | --- |
| rs3757840 | 7:44231216 | T | G | 0.526 | 0.0191 | 0.0032 | 4.52E-10 |
| rs887688 | 7:44185656 | T | G | 0.191 | -0.0224 | 0.0037 | 7.65E-11 |
| rs1799884 | 7:44229068 | T | C | 0.184 | 0.0333 | 0.0034 | 1.58E-23 |

SNPs were extracted from Meta-Analyses of Glucose and Insulin-related traits Consortium. HbA_1c_ was measured in percentage. SNP, single nucleotide polymorphism; EAF, effect allele frequency.

| Exposure | Outcome | Case/control | OR (95% CI) | *P* |
| --- | --- | --- | --- | --- |
| Genetically proxied GK activation (per 1% lower HbA_1c_) instrumented by 3 SNPs | CAD | 29319/183134 | 0.47 (0.28, 0.80) | 0.005 |
|  | HF | 10540/168186 | 0.67 (0.22, 2.08) | 0.493 |
|  | PAD | 3593/208860 | 1.01 (0.27, 3.83) | 0.987 |
|  | Stroke | 17671/192383 | 0.84 (0.44, 1.59) | 0.594 |

Table S6. Associations of genetically proxied GK activation with risks of cardiovascular outcomes in East Asian population.

All estimations were based on the inverse variance weighted method. 1% lower HbA_1c_ equals to 11 mmol/mol lower. OR, odds ratio; CI, confidence interval; GK, glucokinase activation; SNP, single nucleotide polymorphism; CAD, coronary artery disease; HF, heart failure; PAD, peripheral arterial disease.

Table S7. Instrumental variables for non-targeted HbA_1c_ lowering and their associations with HbA_1c_.

| SNP | Position | Effect allele | Other allele | EAF | Effect size | Standard error | P |
| --- | --- | --- | --- | --- | --- | --- | --- |
| rs267738 | 1:150940625 | T | G | 0.797 | 0.0109 | 0.0016 | 1.14E-11 |
| rs857725 | 1:158607935 | T | G | 0.723 | -0.0208 | 0.0014 | 5.43E-55 |
| rs7547793 | 1:203653544 | A | C | 0.12 | -0.0118 | 0.0021 | 6.61E-09 |
| rs340882 | 1:214145731 | C | G | 0.42 | -0.0084 | 0.0013 | 1.48E-10 |
| rs2375278 | 1:25529038 | A | G | 0.176 | 0.0112 | 0.0017 | 1.05E-11 |
| rs1175549 | 1:3691727 | A | C | 0.786 | 0.0098 | 0.0015 | 7.13E-13 |
| rs560887 | 2:169763148 | T | C | 0.306 | -0.0307 | 0.0014 | 5.55E-122 |
| rs13389076 | 2:169789512 | A | G | 0.034 | 0.0332 | 0.0038 | 3.04E-18 |
| rs13419763 | 2:219134950 | T | C | 0.588 | 0.008 | 0.0014 | 5.48E-09 |
| rs12612492 | 2:24093756 | T | C | 0.148 | 0.0188 | 0.0019 | 1.88E-26 |
| rs1367173 | 2:43449385 | T | C | 0.106 | -0.0152 | 0.002 | 1.66E-14 |
| rs79403657 | 2:48114094 | C | G | 0.823 | -0.009 | 0.0017 | 2.03E-08 |
| rs10169706 | 2:5791194 | T | C | 0.04 | 0.026 | 0.0046 | 1.48E-08 |
| rs12491937 | 3:12268244 | A | G | 0.555 | 0.009 | 0.0013 | 1.42E-13 |
| rs11719201 | 3:123068744 | T | C | 0.182 | -0.0129 | 0.0015 | 2.43E-18 |
| rs6804915 | 3:170627909 | A | C | 0.288 | -0.0108 | 0.0014 | 2.76E-16 |
| rs13089972 | 3:171798694 | A | T | 0.584 | 0.0111 | 0.0014 | 1.87E-15 |
| rs9818758 | 3:49382925 | A | G | 0.204 | 0.0131 | 0.0017 | 1.49E-13 |
| rs6798941 | 3:52893465 | T | C | 0.322 | 0.0086 | 0.0015 | 1.49E-08 |
| rs13134327 | 4:144659795 | A | G | 0.331 | 0.0144 | 0.0014 | 2.81E-26 |
| rs6877043 | 5:154048367 | T | C | 0.638 | 0.0085 | 0.0014 | 1.99E-10 |
| rs9376090 | 6:135411228 | T | C | 0.728 | 0.0247 | 0.0014 | 1.90E-62 |
| rs10946402 | 6:20715826 | T | G | 0.831 | -0.0101 | 0.0016 | 1.12E-10 |
| rs1800562 | 6:26093141 | A | G | 0.046 | -0.0383 | 0.0027 | 2.33E-50 |
| rs204995 | 6:32154285 | A | G | 0.781 | -0.0098 | 0.0018 | 1.93E-09 |
| rs3778321 | 6:7250270 | A | G | 0.176 | -0.0106 | 0.0016 | 4.18E-11 |
| rs4727979 | 7:123429697 | A | C | 0.906 | 0.0121 | 0.0024 | 4.61E-08 |
| rs10231021 | 7:15060429 | A | T | 0.492 | 0.0089 | 0.0013 | 8.69E-14 |
| rs2908277 | 7:44183433 | A | G | 0.117 | 0.0166 | 0.002 | 1.29E-18 |
| rs2971670 | 7:44226101 | T | C | 0.181 | 0.0316 | 0.0017 | 5.10E-88 |
| rs13234131 | 7:73025975 | A | G | 0.876 | -0.0113 | 0.002 | 2.06E-09 |
| rs11558471 | 8:118185733 | A | G | 0.707 | 0.0151 | 0.0014 | 3.38E-25 |
| rs2001846 | 8:126478450 | T | C | 0.471 | -0.0069 | 0.0013 | 8.58E-10 |
| rs6474359 | 8:41549194 | T | C | 0.978 | 0.0427 | 0.0038 | 1.91E-33 |
| rs4737009 | 8:41630405 | A | G | 0.262 | 0.0228 | 0.0015 | 8.29E-56 |
| rs7042939 | 9:110511408 | A | G | 0.418 | 0.0102 | 0.0013 | 1.50E-15 |
| rs651007 | 9:136153875 | T | C | 0.215 | 0.0108 | 0.0015 | 3.28E-15 |
| rs3829109 | 9:139256766 | A | G | 0.276 | -0.0086 | 0.0015 | 2.68E-08 |
| rs10811661 | 9:22134094 | T | C | 0.835 | 0.0128 | 0.0017 | 1.74E-14 |
| rs7861647 | 9:79977386 | T | C | 0.193 | 0.0128 | 0.0016 | 4.50E-14 |
| rs61750929 | 9:91495135 | T | C | 0.041 | -0.0284 | 0.0029 | 9.49E-24 |
| rs7903146 | 10:114758349 | T | C | 0.307 | 0.0133 | 0.0014 | 1.04E-22 |
| rs11257655 | 10:12307894 | T | C | 0.241 | 0.011 | 0.0016 | 1.91E-13 |
| rs2102339 | 10:71015389 | T | C | 0.334 | -0.0087 | 0.0014 | 3.42E-10 |
| rs16926246 | 10:71093392 | T | C | 0.136 | -0.0727 | 0.0021 | 1.00E-200 |
| rs7127313 | 11:100508897 | T | C | 0.336 | 0.0066 | 0.0013 | 4.85E-08 |
| rs608793 | 11:118986659 | T | C | 0.479 | 0.0065 | 0.0013 | 4.55E-08 |
| rs4980325 | 11:234451 | T | G | 0.532 | 0.0108 | 0.0014 | 4.70E-14 |
| rs11039154 | 11:47278502 | T | C | 0.277 | -0.0087 | 0.0014 | 3.11E-09 |
| rs174559 | 11:61581656 | A | G | 0.285 | -0.0106 | 0.0014 | 3.31E-13 |
| rs10830963 | 11:92708710 | C | G | 0.714 | -0.0197 | 0.0015 | 1.54E-36 |
| rs360147 | 11:9790817 | T | C | 0.264 | -0.0086 | 0.0015 | 2.08E-09 |
| rs10774624 | 12:111833788 | A | G | 0.525 | 0.0093 | 0.0013 | 4.17E-14 |
| rs117233107 | 12:4328521 | A | G | 0.02 | -0.047 | 0.0072 | 8.45E-11 |
| rs4760682 | 12:48512285 | A | C | 0.817 | 0.0164 | 0.0018 | 3.20E-20 |
| rs76533333 | 13:113352916 | A | G | 0.913 | -0.0265 | 0.0025 | 2.81E-29 |
| rs1278769 | 13:113536627 | A | G | 0.231 | -0.0091 | 0.0015 | 5.52E-12 |
| rs1535464 | 14:100793431 | A | G | 0.212 | -0.0086 | 0.0017 | 1.11E-08 |
| rs151165 | 14:65272626 | A | T | 0.397 | 0.0079 | 0.0014 | 2.04E-09 |
| rs10151436 | 14:73616095 | A | T | 0.89 | 0.013 | 0.0021 | 3.85E-11 |
| rs452306 | 15:65822777 | T | C | 0.627 | -0.0098 | 0.0014 | 5.51E-13 |
| rs11643024 | 16:11443183 | A | G | 0.303 | 0.0084 | 0.0015 | 7.98E-10 |
| rs7190771 | 16:28590030 | A | G | 0.332 | 0.0085 | 0.0013 | 6.02E-11 |
| rs11248914 | 16:293562 | T | C | 0.698 | 0.0114 | 0.0014 | 1.42E-14 |
| rs7198799 | 16:68818390 | T | C | 0.281 | 0.0083 | 0.0014 | 4.76E-09 |
| rs837763 | 16:88853729 | T | C | 0.578 | 0.0176 | 0.0013 | 5.20E-38 |
| rs9914988 | 17:27183104 | A | G | 0.802 | 0.0125 | 0.0016 | 4.66E-17 |
| rs2748427 | 17:76121864 | A | G | 0.803 | -0.0307 | 0.0022 | 9.82E-49 |
| rs9909940 | 17:80689036 | T | C | 0.323 | 0.0322 | 0.0014 | 1.43E-116 |
| rs28671200 | 18:43774444 | T | G | 0.646 | 0.0086 | 0.0017 | 1.56E-08 |
| rs17533945 | 19:17257802 | T | C | 0.582 | -0.0128 | 0.0014 | 1.62E-23 |
| rs10405535 | 19:33072085 | A | G | 0.29 | 0.0122 | 0.0016 | 6.47E-14 |
| rs737092 | 20:55990405 | T | C | 0.501 | -0.0073 | 0.0013 | 7.57E-09 |
| rs855791 | 22:37462936 | A | G | 0.4 | 0.0188 | 0.0013 | 1.34E-56 |
| rs8138197 | 22:43114551 | A | G | 0.488 | -0.0073 | 0.0014 | 3.54E-08 |

SNPs were extracted from Meta-Analyses of Glucose and Insulin-related traits Consortium. The population was restricted to European ancestry. HbA1c was measured in percentage. SNP, single nucleotide polymorphism; EAF, effect allele frequency.

| Exposure | Outcome | OR/Beta (95% CI) | *P* |
| --- | --- | --- | --- |
| Genetically predicted lower HbA_1c_ (per 1% lower HbA_1c_) instrumented by 73 SNPs (without *GCK* variants) | T2D | 0.29 (0.11, 0.79) | 0.015 |
|  | Insulin level | −0.23 (−0.69, 0.23) | 0.324 |
|  | CAD | 0.78 (0.60, 1.02) | 0.073 |
|  | HF | 1.01 (0.81, 1.26) | 0.956 |

Table S8. Associations of genetically predicted lower HbA_1c_ with outcomes after removing GCK variants.

All estimations were based on the inverse variance weighted method. The population was restricted to European ancestry. 1% lower HbA_1c_ equals to 11 mmol/mol lower. OR, odds ratio; CI, confidence interval; SNP, single nucleotide polymorphism; T2D, type 2 diabetes; CAD, coronary artery disease; HF, heart failure.

**Supplementary Figure**


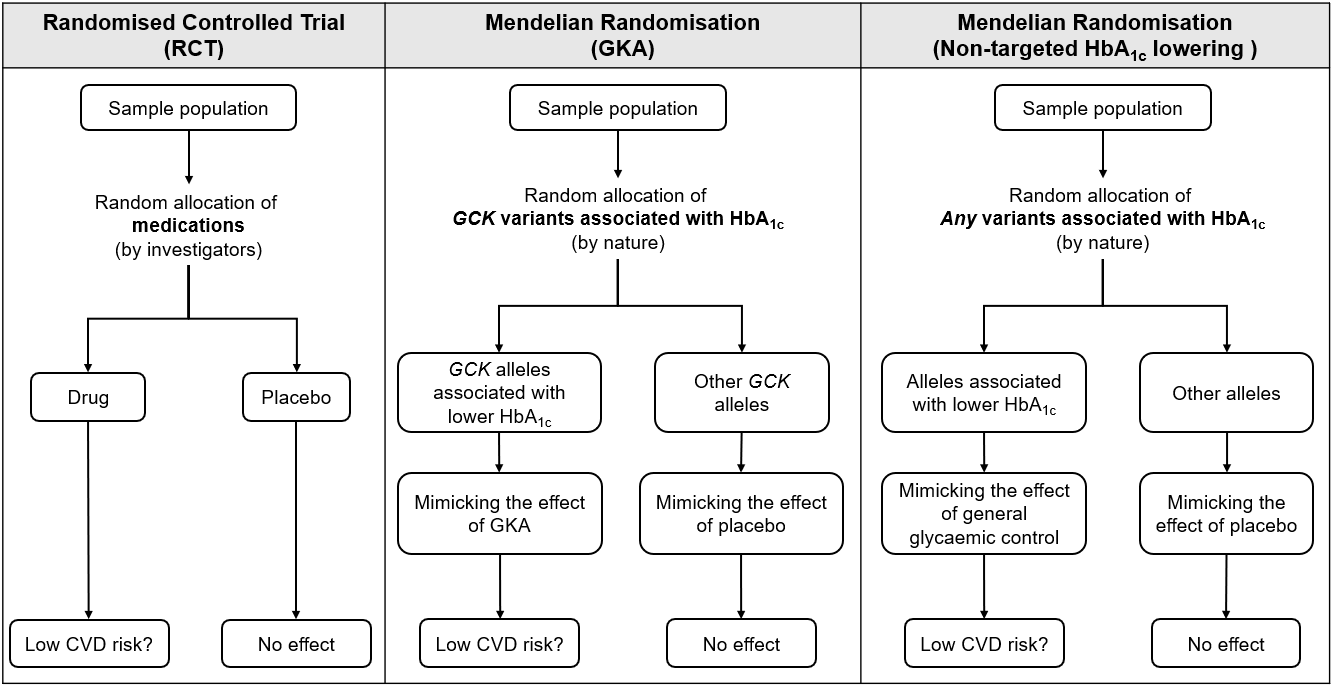


Figure S1. Conceptual framework of study design. In analogy to a conventional randomised controlled trial (left panel), Mendelian randomisation of genetically proxied GK activation (middle panel) as primary analysis and of genetically proxied non-targeted HbA_1c_ lowering (right panel) for comparison. CVD, cardiovascular disease; GKA, glucokinase activator.

**Supplementary Method. Brief summary of outcome definition**

**Coronary artery disease (CAD) (European)**

(https://www.ebi.ac.uk/gwas/studies/GCST005195, PMID: 29212778)

Prevalent and incident cases from UK biobank:

- ICD 10 codes: I21-I25 covering ischemic heart diseases
- Office of Population Censuses and Surveys Classification of Interventions and Procedures version 4 (OPCS-4) codes: K40-K46, K49, K50 and K75 which includes replacement, transluminal balloon angioplasty, and other therapeutic transluminal operations on coronary artery and percutaneous transluminal balloon angioplasty and insertion of stent into coronary artery
- Self-reported CAD: heart attack/myocardial infarction, coronary angioplasty +/- stent, cabg and triple heart bypass

Prevalent and incident cases from CARDIoGRAM Consortium:

| **Study** | **Heart Failure Definition** |
| --- | --- |
| *ADVANCE* | Clinical non-fatal CAD (men ≤45 yrs, women ≤55 yrs) including AMI (enzymes), typical angina with ≥1 artery with >50% stenosis, positive non-invasive test, or PCI or CABG |
| *CADomics* | CAD: >50% stenosis in 1 major coronary artery and/or MI based on ECG and enzymes |
| *CHARGE* | CHD: definite or probable MI, PTCA or CABG, or ECG MI |
| *deCODE* CAD | MI: MONICA criteria (<75 yrs) or discharge diagnosis of MI; CAD: PCI or participation in CVD genetics program with self-report of CABG or PCI, or discharge diagnosis of angina pectoris, MI or chronic ischaemic heart disease |
| *GERMIFS* *I* | MI (<65 yrs) with >1 1st degree sibling with severe CAD (PTCA; MI; CABG) |
| *GERMIFS* *II* | MI (<60 yrs); 59.4% with family history of CAD |
| *GERMIFS* *III (KORA)* | MI (<60 yrs); MONICA criteria |
| *LURIC/ AtheroRemo 1* | Symptoms of angina pectoris, NSTEMI, STEMI, or >50% coronary stenosis |
| *LURIC/ AtheroRemo 2* | Symptoms of angina pectoris, NSTEMI, STEMI, or >50% coronary stenosis |
| *MedStar* | Angiography (≥1 coronary vessel with >50% stenosis); ≤55 for males and ≤60 for females. |
| *MIGen* | MI (men <50 yrs / women <60 yrs) |
| *OHGS1* | Angiographic (>50% stenosis) |
| *PennCATH* | Angiography (≥1 coronary vessel with >50% stenosis); ≤60 for males and ≤65 for females. |
| *WTCCC* | Validated MI, CABG, PTCA or angina with positive non-invasive testing <66 yrs |

**Heart failure (HF) (European)**

(Heart Failure Molecular Epidemiology for Therapeutic Targets (HERMES) Consortium, https://www.hermesconsortium.org/, PMID: 29212778)

Prevalent and incident cases from following cohorts:

| **Study** | **Heart Failure Definition** |
| --- | --- |
| ARIC | Incident HF: the first HF hospitalization or presence of HF code on death certificate since baseline visit through 2013. ICD-9 code 428.x, and deaths with ICD-9/10 codes of either 428.x or I50.  Prevalent HF: ascertained at first visit. If the participant reported to have taken any medication for heart failure, or qualifies for the Gothenburg Criteria. |
| BIOSTAT-CHF | Based on physician diagnosis, previous documented admission with heart failure requiring diuretic treatment, treatment with furosemide ≥20 mg/day or equivalent. |
| CHS | Identified by self-report or administrative data validated by physician’s review of medical records, as described in previous reports (PMID: 1669507). |
| COGEN | Inclusion criteria: patients ≥18 years, LVEF <40% or symptoms of clinical HF assessed by a physician including NYHA>1 Exclusion criteria: patients with cardiac valvular pathology (e.g. aortic stenosis), HTx, PAH or other structural heart disease |
| deCODE | ICD-10: I50 and subcodes, ICD-9: 428 and subcodes |
| EGCUT | ICD-10: I50 and subcodes. |
| EPHESUS | The presence of pulmonary rales, chest radiography showing pulmonary venous congestion, or the presence of a third heart sound. |
| EPIC-Norfolk | ICD-10: I50 and subcodes |
| FHS | Criteria for defining heart failure in the FHS have been described previously (PMID: 5122894, 16837677). |
| FINRISK | ICD-10: I50, I110, I130 and I132; ICD-9: 4029B, 404, 4148, 428; ICD-7: 42700, 42710, 428 or special drug reimbursement for heart failure medications (requires a medical certificate that meets predefined criteria for heart failure). |
| GoDARTS | Echocardiographic evidence of left ventricular systolic impairment and diuretic prescription or admission to hospital with HF and receipt of a loop diuretic prescription |
| GRADE | Patients who were ≥18 years of age with a diagnosis of at least moderate systolic left ventricular dysfunction (EF ≤30%), and who had an ICD at the University of Pittsburgh Medical Center. |
| LURIC | The combined presence of symptoms of dyspnea on exertion and cardiac disease with impaired left ventricular function. |
| MDCS | Diagnosis codes 427.00, 427.10, and 428.99 for ICD-8, 428 ICD-9, and I50 and I11.0 for the ICD-10 as primary diagnosis, according to a previous validation study (PMID:15916919). |
| PHFS | Diagnosed by a heart failure cardiologist based on clinical evaluation and cardiac imaging |
| PIVUS | ICD codes 427.00, 427.10, 428 (ICD-9), I50 (ICD-10) and hypertensive heart disease with heart failure, I11.0 (ICD-10) PMID:15916919. |
| PREVEND | Heart failure cases were ascertained using criteria in accordance with the Heart Failure Guidelines of the European Society of Cardiology (ESC). |
| PROSPER | Based on a combination of symptoms and signs, including chest radiograph with fluid congestion or echocardiogram with severely diminished LV function. |
| Regeneron/Geisinger | ICD-10: I50 and subcodes. |
| Rotterdam study 1 | Prevalent heart failure at baseline was assessed using a validated score based on the European Society of Cardiology recommendation, identified from hospital discharge diagnoses, and restropective medical records screening. Cases of incident heart failure were obtained by continuously monitoring participants for the occurrence of heart failure during follow-up through general practitioners’ records and hospital discharge diagnoses. |
| SHIP | According to a modified Rotterdam definition (PMID: 10213348). |
| SOLID | Heart failure status at enrolment was identified from medical record with no specific definition. HF during follow up were defined as admission to hospital or attendance at an acute health care facility for administration of intravenous diuretic treatment, escalation of diuretic doses, and/or inotropes. |
| TwinGene | Based on ICD-10: I50; ICD-8 and ICD-9 428 |
| UK Biobank | Individuals with self-reported "HF/pulmonary edema" or "cardiomyopathy"; or ICD-10: I11.0, I13.0, I13.2, I25.5, I42.0, I42.5, I42.8, I42.9, I50.0, I50.1, I50.9; ICD-9: 4254, 4280, 4281, 4289. |
| ULSAM | ICD codes 427.00, 427.10, 428 (ICD-9), I50 (ICD-10) and hypertensive heart disease with heart failure, I11.0 (ICD-10) as possible diagnosis of heart failure. |
| WGHS | Heart failure cases were ascertained by cardiologists from medical records. |

**Stroke (European)**

(MEGASTROKE consortium, https://www.megastroke.org/, PMID: 29531354)

Prevalent and incident cases from following cohorts:

| **Study** | **Heart Failure Definition** |
| --- | --- |
| *ASGC* | Stroke was defined by WHO criteria as a sudden focal neurologic deficit of vascular origin, lasting more than 24 hours and confirmed by imaging such as computerised tomography (CT) and/or magnetic resonance imaging (MRI) brain scan. Other investigative tests such as electrocardiogram, carotid doppler and transoesophageal echocardiogram were conducted to define IS mechanism as clinically appropriate. |
| *BRAINS* | Diagnosis of stroke was confirmed using positive imaging (MRI or CT) and ischemic stroke subtypes were assigned using TOAST criteria, based on clinical, imaging and risk factor data. |
| *GEOS* | Cases were identified through discharge surveillance from 59 participating hospitals and direct physician referral from a defined geographic region. |
| *HPS* | Individuals entering HPS with a clinical diagnosis of ischemic stroke were used as cases in the METASTROKE study. |
| *ISGS* | All recruits were extensively clinically phenotyped and have imagingconfirmed ischemic stroke using either CT or MRI brain scans. Probands are adult men and women over the age of 18 years diagnosed with ischemic stroke confirmed by a study neurologist on the basis of history, physical examination and CT or MR imaging of the brain who also have a history of at least one living sibling with a history of stroke. |
| *MGH-GASROS* | Ischemic stroke was defined as either (1) a radiographically proven (head CT or MRI) infarct associated with the appropriate clinical stroke syndrome, or (2) a fixed neurological deficit persisting more than 24 hours, consistent with a vascular pattern of involvement and without radiographic evidence of demyelinating disease, or other non-vascular structural disease. |
| *Milano* | Ischemic stroke cases, first ever or recurrent, confirmed on brain imaging, were selected for this study. All cases were of selfreported Caucasian ancestry and had clinically relevant diagnostic workup performed. All cases were phenotyped by an experienced stroke neurologist according to TOAST criteria, based on relevant clinical imaging and available information on cardiovascular risk factors. |
| *WTCCC2* | All cases were of self-reported Caucasian ancestry. Ischemic stroke subtypes were determined according to TOAST criteria based on relevant clinical imaging and available information on cardiovascular risk factors. |
| *VISP* | Nondisabling cerebral infarction was defined as an ischemic brain infarction not due to embolism from a cardiac source, characterized by the sudden onset of a neurological deficit. |
| *WHI* | Stroke diagnosis requiring and/or occurring during hospitalization was based on rapid onset of a neurological deficit attributable to an obstruction or rupture of an arterial vessel system. Hospitalized incident stroke events were identified by semiannual questionnaires and adjudicated following medical record review, which occurred both locally and centrally. |
| *BASICMAR* | Ischemic stroke etiologic subtypes were classified according to TOAST criteria. |
| *GRAZ* | Ischemic stroke was defined as an episode of focal neurological deficits with acute onset and lasting > 24 hours. |
| *KRAKOW* | All cases were phenotyped independently by two experienced stroke neurologists with review of original imaging. Cases were subsequently classified additionally using the CCS system. |
| *LSGS* | Cases of European descent with cerebral ischemia, defined as a clinical stroke with imaging confirmation or a TIA with a new ischemic lesion on diffusion-weighted imaging. |
| *LSR* | Stroke was defined using the WHO criteria.12 Subjects aged 18 years or older with stroke caused by cerebral infarct, intracerebral hemorrhage or subarachnoid hemorrhage are included. |
| *MCISS* | All subjects with clinical suspicion of a stroke were admitted through the emergency room to a dedicated stroke unit supervised by a vascular neurologist. |
| *MIAMISR* | an ongoing prospective hospital registry of consecutive patients subjects with prevalent stroke (ischemic and hemorrhagic) and TIA with available neuroimaging (CT or MRI) who provide informed consent. |
| *NHS* | prospectively identified incident strokes and confirmed ischemic stroke cases by medical record review. Clinical symptoms consistent with stroke and exclusion of alternate etiologies were required for classification of stroke. |
| *NOMAS* | First-ever ischemic stroke cases were identified for the casecontrol study by screening of patient admissions, discharge codes, and referrals for neuroimaging at 15 acute care hospitals in the defined study area and multiple approaches to monitor for non-hospitalized cases. Incident ischemic stroke cases were identified from the prospective cohort study through follow-up visits and scheduled telephone contacts. |
| *REGARDS* | A symptom-based approach, independent of neuroimaging outcome, is used to confirm events using the WHO definition of stroke. |
| *SPS3* | Principal eligibility criteria include man or woman at least 30 years of age with clinical evidence of small subcortical stroke and brain MRI evidence of small subcortical infarct. |
| *WUSTL* | Subjects were retained in the study if their discharge diagnosis was ischemic stroke (without requirement for the stroke to be visualized on CT or MRI). |
| *AGES* | Incident stroke cases were ascertained from multiple sources including hospital, general practice, nursing home records and death certificates. All possible cases were adjudicated with standard TOAST criteria by two Neurologists and a Neuroradiologist with expertise in evaluating stroke cases for epidemiologic studies. |
| *CHS– European Ancestry* | Stroke definitions were derived from the criteria used for the Systolic Hypertension in the Elderly Program (SHEP). |
| *FHS* | Incident strokes have been identified since 1948 through this ongoing system of FHS clinic and local hospital surveillance; they include review of medical records and collaboration with local general practitioners, emergency rooms and imaging facilities. |
| *FINRISK* | ICD-codes: I63; not I63.6, I64 (ICD-10) / 4330A, 4331A, 4339A, 4340A, 4341A, 4349A, 436 (ICD-9) / 433, 434, 436 (ICD-8) for Ischemic stroke excluding any hemorrhagic strokes, and I60-I61,I63-I64 (not I63.6) (ICD-10) / 430, 431, 4330A, 4331A, 4339A, 4340A, 4341A, 4349A, 436 (ICD-9) / 430, 431 (except 431.01, 431.91), 433, 434, 436 (ICD-8) for allstroke including SAH. ICD-8 codes 430, 431 (excluding codes 431.01, 431.91 of the Finnish adaptation of ICD-8*), 432, 433, 434 or with ICD-9 codes 430, 431, 433 (excluding codes 4330X, 4331X, 4339X of the Finnish adaptation of ICD-9*), 434 (excluding code 4349X of the Finnish adaptation of ICD-9*), 436, 437, 438 or with ICD-10 codes I60, I61, I63 (excluding I63.6), I64 orI69 |
| *Health ABC* | Participants were screened for stroke events every 6 months alternating between semi-annual phone interviews and annual clinical visits. Any self-reported hospitalization for stroke led to medical record abstraction and verification by a Health ABC Disease Adjudicator at each site. |
| *Rotterdam* | For suspected stroke and TIA events, both fatal and non-fatal, additional information (including neuroimaging) was obtained from general practitioner’ and hospital records and research physicians discussed available information with an experienced stroke neurologist to verify all diagnoses and to subclassify the strokes. |
| *SHIP* | For in- and outpatient data any stroke was defined as cases with a coded ICD I61, I63, I64, I69.1, I69.3, I69.4 diagnosis. For ischemic stroke we included all cases with I63.x codes based on in- and outpatient data. |
| *WGHS* | A confirmed stroke was defined as a new neurologic deficit of sudden onset that persisted for >24 h. Clinical information as well as computed tomographic scans or MRI were used to distinguish hemorrhagic from ischemic events. |
| *MESA* | To verify self-reported diagnoses, information was collected from death certificates and medical records for all hospitalizations and outpatient cardiovascular diagnoses, using ICD-9 and ICD-10 codes. |
| *TWINGENE* | For stroke, the following ICD codes were used: ICD-8 codes 430–436, ICD-9 codes 430–436 and ICD-10 codes I60-I64 and G45.52 Further classification into stroke subtypes was done using ICD-8 codes 432-434, ICD-9 codes 433-434, and ICD-10 code I63 for ischemic stroke, and ICD-8 codes 430-431, ICD-9 codes 430-432, and ICD-10 codes I60-I62 for hemorrhagic stroke. |
| *ULSAM* | ICD-8 codes 430-431 and 433-434, ICD-9 codes 430-432, 434 or ICD-10 codes I60-I64. |
| *3C-Study* | Stroke was confirmed if the participant had a new focal neurological deficit of sudden onset attributable to a cerebrovascular event that persisted for more than 24 hours. |
| *EPIC* | Stroke events were defined by ICD10 codes as follows: Ischemic I63, Haemorrhagic I61, SAH I60, Unclassified I64, Other CRBV I62, I65- I69, F01. |
| AIDHS/SDS | Diagnosis of ischemic stroke was established based on either evidence of an infarction in neuroimaging (CT/MRI scan) or symptom duration >24 hours. |
| *VHIR-FMT-Barcelona* | Cases were selected through demonstration of acute ischemic stroke in a neuroimaging study during the first 7 days after stroke. |
| *CADISP* | patients with an ischemic stroke without cervical artery dissection (non-CeAD ischemic stroke) were recruited |
| *ARIC* | all local hospitals annually provided lists of stroke discharges (International Classification of Diseases, Ninth Revision, Clinical Modification codes 430 to 438), which were scrutinized for ARIC participant discharges. |
| *JHS* | The definition of stroke was based on the World Health Organization (WHO) criteria for definition of stroke or clinical criteria in which case the WHO criteria might not have been satisfied, but there is clinical evidence sufficient for a diagnosis of stroke to be made. |
| *Helsinki 2000 Ischemic Stroke Genetics Study* | Only patients with positive neuroimaging findings for a newonset brain infarction were recruited following written informed consent. |
| *Hisayama-FSR* | Ischemic stroke was defined as a sudden nonconvulsive, focal neurologic deficit lasting longer than 24 hours due to brain ischemia. The diagnoses of ischemic stroke and its subtypes for all cases were made by stroke neurologists of the hospitals, referring to detailed clinical features and ancillary laboratory examinations. |
| *HVH 1 & 2* | Ischemic stroke cases satisfied one or more of the following criteria: (a) Focal deficit, without evidence of blood on CT or MRI, (b) Focal deficit, with mottled appearance in the appropriate location on CT, or (c) surgery or autopsy evidence of infarction. |
| *INTERSTROKE* | cases were stroke patients with acute first stroke (within 5 days of symptoms onset and 72 hours of hospital admission) in whom neuroimaging (CT or MRI) was performed. Stroke was defined with the WHO clinical criteria for stroke. |
| *MDC* | Criteria for stroke was rapidly developing clinical signs of local or global loss of cerebral function lasting for >24 hours or leading to death before then, with no apparent cause other than cerebral ischemia or hemorrhage. |
| *RACE* | Cases were eligible for inclusion in the study if they: (1) are aged at least 18 years; (2) presented with a sudden onset of neurological deficit affecting a vascular territory with sustained deficit at 24 hours verified by medical attention within 72 hours after onset (onset is defined by when the patient was last seen normal and not when found with deficit); (3) the diagnosis was supported by CT/MRI; and (4) presented with a Modified Rankin Score of < 2 prior to the stroke. |
| *SAHLSIS* | Inclusion criteria was ischemic stroke which was defined as an episode of focal neurological deficits with acute onset and lasting > 24 hours or until death, with no apparent non-vascular cause, and no signs of primary hemorrhage on brain imaging. |
| *SIFAP* | First-ever (80.5%) and recurrent ischemic strokes were included. MRI was a mandatory procedure but, in the case of negative or missing MRI, a qualified stroke neurologist could confirm the clinical diagnosis |
| *SLESS* | One consultant neurologist performed stroke subtyping using data collected on a standard proforma with additional review of all original brain imaging in all patients, as well as review of original notes when necessary. |
| *UK - young lacunar stroke DNA resource* | Lacunar stroke was defined as a clinical lacunar syndrome, with an anatomically compatible lesion on MRI (subcortical infarct ≤15 mm in diameter). |
| *ICH* | Cases were ascertained across participating studies according to predefined standardized criteria. Spontaneous ICH was defined as a new and acute neurological deficit with compatible brain imaging (computed tomography or magnetic resonance imaging) showing the presence of intraparenchymal bleeding. |

**Peripheral arterial disease (PAD) (European)**

(FinnGen Consortium data freeze 2, https://www.finngen.fi/en/researchers/clinical-endpoints)

PAD cases are defined as ICD 10th codes E105, E115, E125, E135, E145, I702 and I739; ICD 9th codes 4402 and 4439; ICD 8th codes 25006, 4402 and 4439.
